# Probing Substrate/Catalyst Effects Using QSPR Analysis on Friedel-Crafts Acylation Reactions over Hierarchical BEA Zeolites

**DOI:** 10.3390/molecules25235682

**Published:** 2020-12-02

**Authors:** Ruben Elvas-Leitão, Filomena Martins, Leonor Borbinha, Catarina Marranita, Angela Martins, Nelson Nunes

**Affiliations:** 1 Área Departamental de Engenharia Química, Instituto Superior de Engenharia de Lisboa, IPL, R. Conselheiro Emídio Navarro, 1959-007 Lisboa, Portugal; amartins@deq.isel.ipl.pt; 2 Centro de Química Estrutural (CQE), Faculdade de Ciências, Universidade de Lisboa, 1749-016 Lisboa, Portugal; filomena.martins@ciencias.ulisboa.pt (F.M.); leonor.b.97@hotmail.com (L.B.); 3 Escola Profissional de Setúbal, R. Professor Borges de Macedo, n° 1, 2910-001 Setúbal, Portugal; cmarranita.9660@alunos.eps.pt

**Keywords:** QSPR analysis, hierarchical BEA, Friedel-Crafts acylation, kinetic modelling, nonlinear regression

## Abstract

Attempts to optimize heterogeneous catalysis often lack quantitative comparative analysis. The use of kinetic modelling leads to rate (*k*) and relative sorption equilibrium constants (*K*), which can be further rationalized using Quantitative Structure-Property Relationships (QSPR) based on Multiple Linear Regressions (MLR). Friedel-Crafts acylation using commercial and hierarchical BEA zeolites as heterogeneous catalysts, acetic anhydride as the acylating agent, and a set of seven substrates with different sizes and chemical functionalities were herein studied. Catalytic results were correlated with the physicochemical properties of substrates and catalysts. From this analysis, a robust set of equations was obtained allowing inferences about the dominant factors governing the processes. Not entirely surprising, the rate and sorption equilibrium constants were found to be explained in part by common factors but of opposite signs: higher and stronger adsorption forces increase reaction rates, but they also make the zeolite active sites less accessible to new reactant molecules. The most relevant parameters are related to the substrates’ molecular size, which can be associated with different reaction steps, namely accessibility to micropores, diffusion capacity, and polarizability of molecules. The relatively large set of substrates used here reinforces previous findings and brings further insights into the factors that hamper/speed up Friedel-Crafts reactions in heterogeneous media.

## 1. Introduction

Quantitative Structure-Property Relationships (QSPR) are currently applied to an ever-growing plethora of physicochemical processes, be it to predict new data, rationalize phenomena, or both. In this study, Multiple Linear Regression (MLR) is applied to a heterogenous catalytic reaction to study which changes in the substrates’ characteristics (size, polarity, etc.) affect the kinetic and equilibrium properties of the system. Among other reasons, the quest to rationalize heterogeneous reactions producing the same desired product as their homogeneous counterparts is undoubtedly important, since, for instance, syntheses of active pharmaceutical ingredients (APIs) through acylation reactions such as Friedel-Crafts (FC), generally performed homogeneously with soluble catalysts like AlCl_3_ or HF, can also be carried out through heterogenous catalysis with much less environmental impact.

Despite its high efficiency, FC homogeneous reactions also suffer from serious drawbacks such as the need to work at high temperatures and the use of over stoichiometric amounts of catalysts that cannot be regenerated at the end of the production cycle. The latter, in turn, leads to complicated end-product purifications, often resulting in toxic and corrosive disposal issues [1,2].

Solid acid materials, such as zeolites, are widely used as catalysts in refining and petrochemical processes, since they can be easily separated from the reaction media and reused. However, their use in liquid phase reactions to produce fine chemicals and pharmaceutical products on a large scale is still limited, in spite of some studies reported in the last few years [3,4,5,6,7]. One of the major limitations currently described in the literature is the presence of large molecules that cannot easily access the active sites located inside the microporous structure of zeolites, leading to low catalytic activities. To overcome this limitation, the use of hierarchical zeolites, comprising mesopores in addition to the original microporosity, has been reported with promising results for several zeolite structures. Focusing on liquid-phase Friedel-Crafts acylation reactions, Yan et al. [8] concluded that nano-hierarchical BEA and MFI zeolites showed higher catalytic activity when compared with conventional zeolites. Wang et al. [9] studied the effect of post synthesis treatment on BEA structure, comprising basic and acid treatments. The catalytic behavior was studied using substrates of different sizes, and the performance of hierarchical BEA in the presence of larger molecules was shown to be enhanced. Recently, Linares et al. [10] reported the use of surfactant-templated hierarchical USY as a catalyst in the Friedel-Crafts alkylation of indole using alcohols as acylating agents, and found a significant increase in both activity and reusability, when compared with other catalysts, namely commercial USY zeolite, mesoporous Al-MCM-41, and Amberlyst resin. On the other hand, Cirujano et al. [11] investigated the behavior of Brönsted and Lewis acid sites embedded in ionic liquid Bu_4_PBr and HY zeolite in the alkylation of indole with 4-methoxy-α-methylbenzyl alcohol at 50 °C and found high performance and reusable catalysts. The authors also showed a correlation between the acid strength and catalytic activity, with high turnover frequencies upon five reuse cycles, under mild reaction conditions.

Our quantitative approach aimed at finding and quantifying the combined effect of the properties of both catalyst and substrate molecules, in line with earlier studies already published by us [12,13]. In the first of these studies, the catalytic behavior of hierarchical MWW, a layered commercial zeolite (structure prepared by alkaline and alkaline + acid treatments), was explored using small molecules as substrates (furan, anisole, and pyrrole) and acetic anhydride as the acylating agent [12]. Using kinetic modelling, the authors found enhanced catalysis in the presence of furan and anisole, especially at short reaction times. It was also found from the QSPR analysis that the Brönsted acidity of the hierarchical zeolites was the dominant property, whereas parameters related to dipolarity and Lewis acidity were the most relevant substrate characteristics. Later, the same experimental methodology was followed to prepare hierarchical BEA and among other findings, it was noticed that the Si/Al ratio of the parent zeolites strongly influenced the catalytic behavior of the same set of substrates [13]. These two studies allowed for some quantification of the influence of a few molecular properties, leading to a better understanding of the catalytic and adsorption process involved in these Friedel-Crafts acylation reactions. However, some descriptors used for the substrates were not the most suitable ones for the proposed goal, since among the properties collected at the time, some referred to the behavior of these substances when used as solvents (e.g., *E*_T_). Moreover, QSPR analysis was performed with small substrate molecules that can be considered as model molecules, but with low interest as APIs.

In the present study, the number of substrates was increased, and now comprises additional molecules with different chemical functionalities and larger sizes, in order to significantly enlarge the chemical space under investigation. Additionally, further descriptors/parameters were included to account for the higher diversity and complexity of the substrates’ molecules. The whole set of parameters used correspond to the macroscopic physicochemical properties of the zeolites and of the substrates.

## 2. Results and Discussion

### 2.1. Materials Characterization

The structural and textural properties of the zeolite samples as well as their acidity characterization were presented and discussed in detail elsewhere [13]. However, to facilitate the discussion, and since several of them will be used in the QSPR analysis, the main properties of the catalysts are presented in Table 1 and briefly commented on.

The crystallinity of BEA samples is reduced upon desilication and a small decrease is also verified after acid treatment. Nevertheless, all samples kept the diffraction patterns typical of a BEA structure. Elemental analysis shows, as expected, a decrease in Si/Al ratio as a consequence of Si extraction during the alkaline treatment and, on the other hand, an increase in Si/Al after the acid treatment, indicating the removal of extra-framework debris formed due to desilication. The values corresponding to the textural properties, studied through N_2_ adsorption-desorption isotherms, show a decrease in the microporous volume as well as the expected mesoporosity development because of the alkaline treatment. Upon acid washing with HCl, while the mesoporous volume is kept unchanged, there is a recovery of the microporous volume, denoting the unblocking of the micropores by extra-framework species formed during the alkaline treatment.

The concentration of Brønsted and Lewis acid sites was estimated by integration of FTIR spectra of chemisorbed pyridine onto the acid sites. As can be observed from the data in Table 1, sample BEA-D presents a decrease in both [B]_pyr_ and [L]_pyr_ that can be attributed to partial occlusion of the pore apertures due to some fragment accumulation as well as to some loss of acid sites as a consequence of the alkaline treatment [14,15]. Acid treatment with HCl led to a slight increase in [B]_pyr_ and a decrease in [L]_pyr_ is noticed. This behavior can be attributed to the removal of extra-framework Al species, causing a decrease in [L]_pyr_ and, on the other hand, that removal improves the access of pyridine to the Brønsted acid sites located inside the porous structure, thus increasing [B]_pyr_.

### 2.2. Catalytic Tests

**Figure 1:**
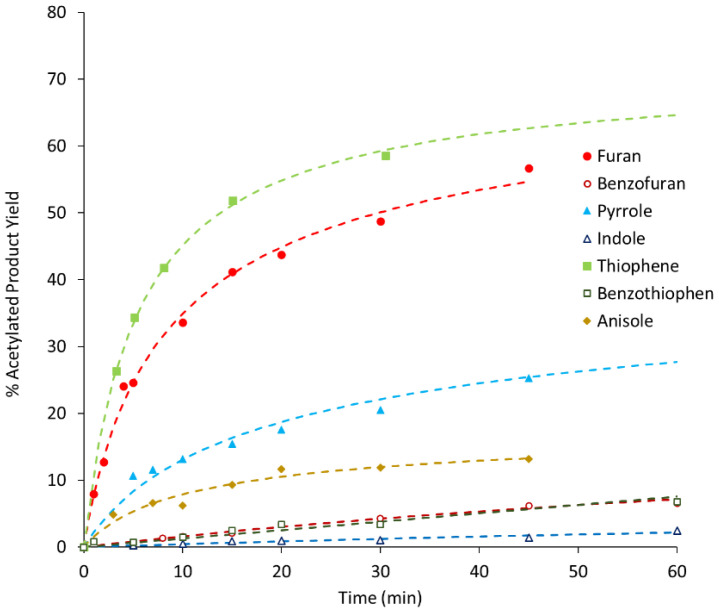
Product yield as a function of reaction time using BEA as the catalyst (the dashed curves represent calculated values resulting from the application of the kinetic model).

**Figure 2:**
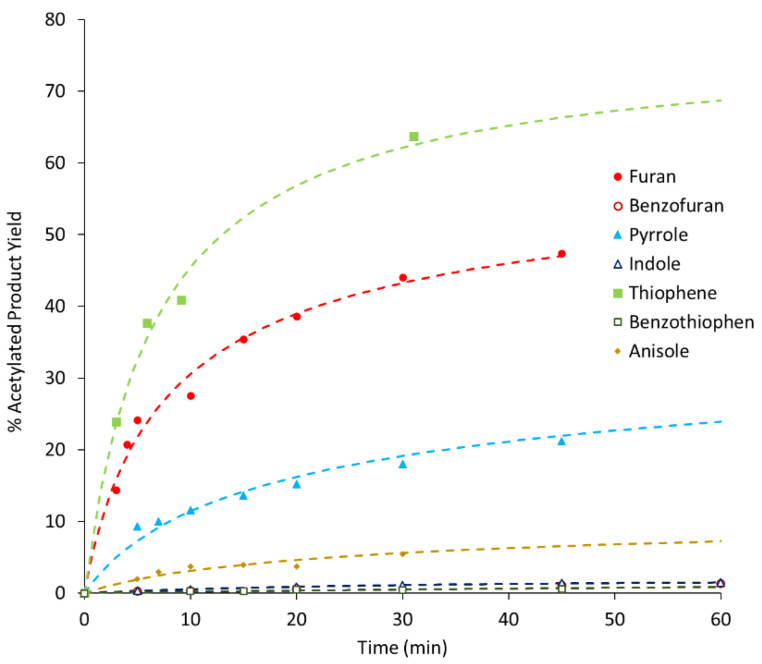
Product yield as a function of reaction time using BEA-D as the catalyst (the dashed curves represent calculated values resulting from the application of the kinetic model).

**Figure 3:**
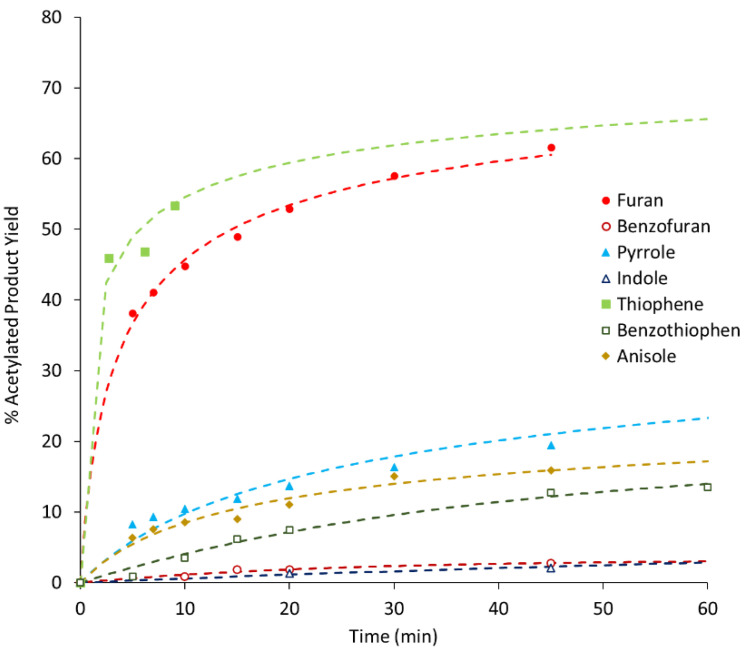
Product yield as a function of reaction time using BEA-D-AT as the catalyst (the dashed curves represent calculated values resulting from the application of the kinetic model).

As stated before, benzofuran, thiophene, benzothiophene, and indole were used as substrates in this work but the catalytic tests concerning furan, pyrrole, and anisole, although already presented in a previous work [13], are herein included to enable QSPR analysis and for comparative purposes. Figure 1, Figure 2 and Figure 3 show the variation with time of product yields in the three different zeolites samples (BEA, BEA-D, BEA-D-AT).

Product yields were calculated in terms of the acylated products that are always obtained in larger amounts. For instance, in the case of furan, the reaction is almost completely selective, leading to 2-acethylfuran due to the high stability of the α-intermediate. On the other hand, in the case of pyrrole, two isomer products are detected due to a lower difference in activation hardness between α and β-intermediates [4]. The presence of additional products is sometimes detected in very small amounts and can be attributed to the occurrence of secondary reactions inside the porous structure of the zeolite samples.

All kinetic curves show a sharp increase in product yield in the first 10 min of reaction, with different rates for different substrates, followed by a slope attenuation. A quick assessment of Figure 1, Figure 2 and Figure 3 shows a clear connection between the size of the substrate molecules (benzofuran, benzothiophene, and indole) and their lower reactivity order. Size restrictions/porosity access seem to play a major role in the reactivity; however, as seen in a previous work [13], other factors are at play. This is particularly visible in the differences among small substrates but with similar sizes (furan, thiophene, and pyrrole). These factors may be related to the interaction of the substrate or product molecules with the catalyst surface.

Overall, the comparison of results among the three catalyst samples shows only slight differences. The textural and chemical modifications performed on treated zeolite samples do not show, at first glance, major differences in the reactivity order and magnitude. Only a mathematical analysis of these curves may unveil further information.

### 2.3. Kinetic Modelling

The simplified form of the Langmuir-Hinshelwood model (Equation (4)) was applied, and nonlinear regression parameters obtained for each substrate/catalyst combination are presented in Table 2, Table 3 and Table 4.

A first analysis of Table 2, Table 3 and Table 4 shows that all nonlinear regressions resulted, in general, in high *R*^2^ and *F* values and low *s_fit_*.

Rate constants obey the following order:

BEA: thiophene > furan > pyrrole > anisole > benzofuran > benzothiophene > indole;

BEA-D: thiophene > furan > pyrrole > anisole > indole > benzofuran > benzothiophene;

BEA-D-AT: thiophene > furan > anisole ≅ pyrrole > benzothiophene > benzofuran > indole.

The data show that the rate constants of thiophene and furan are significantly higher than those for other substrates. However, the numbers do not show a clear pattern when one compares ln *k* or ln *K*_r_ values among the three zeolites. Only a decomposition into significant multiple effects impacting on these properties using a QSPR methodology may provide insight on these processes.

### 2.4. QSPR Analysis

The developed models were based on the correlation of ln *k* and of ln *K*_r_, with the set of seven descriptors related to the zeolite properties presented in Table 1 and the seven substrate properties shown in Table 5, namely: surface area (*σ*), van der Waals volume (*V*_vdW_), density (*ρ*), molar volume (*V*_m_), dipole moment (*μ*), viscosity (*η*), and surface tension (*γ*).

The use of such a high number of descriptors was only possible using, as referred above, an in-house Visual Basic programmed macro for Microsoft Excel to perform a total of 3474 regressions, using all combinations from one to five descriptors. This macro automated several tasks, namely the calculation of the intercorrelation between parameters. These intercorrelations can be seen in Table 6. Values of the determination coefficients, *r*^2^, higher than 0.5 are presented in bold. These descriptors must not be used simultaneously on the fitted models to avoid redundancy. It is also interesting to notice that there is absolutely no numerical correlation between the zeolite descriptors and any of the substrate ones and, therefore there is no need to consider cross terms between zeolite and substrate.

In addition to performing the regressions of all multiple combinations between the 14 descriptors, the Macro allows descriptors’ standardization and the removal of data points considered outliers, i.e., points that are distanced more than two standard deviations from the regression line. The user can also choose the maximum number of descriptors. In the present case, at the start of the analysis, models were limited to a maximum of five descriptors to ensure that one has at least four data points per descriptor.

MLR results were filtered according to a sequence of criteria applied as follows: (a) for each descriptor, only regression coefficients showing a confidence interval ≥ 95% were retained; (b) only equations for which *R*^2^_adj_ ≥ 0.95 were selected; (c) only model equations with two or less outliers were chosen; (d) all equations for which descriptors’ pairwise determination coefficient, *r*^2^, exceeded 0.5 were discarded; (e) only model equations with a top 50% Fisher-Snedecor *F*-value were kept. This procedure led, both for ln *k* and ln *K*_r_, to several, almost statistically identical model equations, but in both cases, it was possible to determine the preferred one using also as criteria, the standard deviation of the fit, *s_fit_*, the adjusted determination coefficient, *R*^2^_adj_, and the predicted determination coefficient, *R^2^*_pred_, to check the internal predictability of each model equation. *R*^2^_pred_ = 1 − *PRESS/SST*, where $PRESS=\sum_{i=1}^{n}{\left(y_{i}-\hat{y}\left(i\right)\right)}^{2}$, $SST=\sum_{i=1}^{n}{\left(y_{i}-\bar{y}\right)}^{2}$, and *ŷ*(i) are the predicted values of the response of the *i*^th^ observation using a model whose estimate is based on the (n−1) data points excluding the *i*^th^ data point. If *R*^2^_adj_ − *R*^2^_pred_ < 0.2, the model shows good predictability. 

Finally, solutions for which coefficients lacked physical meaning were also excluded.

For the rate constant *k*, the chosen equation is the following:

(1) $$\begin{array}{ccccc} \ln k=\; & \left(1.06\;\pm \;0.07\right)- & \;\left(0.76\;\pm \;0.05\right)V_{\mathrm{vdW}}\;- & \;\left(0.52\;\pm \;0.07\right)\rho \;+ & \;\left(0.14\;\pm \;0.04\right){\left[B\right]}_{\mathrm{pyr}}\\ & \left(100\%\right) & \left(100\%\right) & \left(100\%\right) & \left(100\%\right) \end{array}$$

*N* = 21; *R*^2^ = 0.928; *R*^2^_adj_ = 0.915; *R*^2^_pred_ = 0.907; *s_fit_* = 0.08; *F* = 73

The percentage confidence interval of each descriptor is given within round brackets. *R*^2^_pred_ is quite close to *R*^2^_adj_, and therefore, the model has good predictive ability within the covered variable’s space.

**Figure 4:**
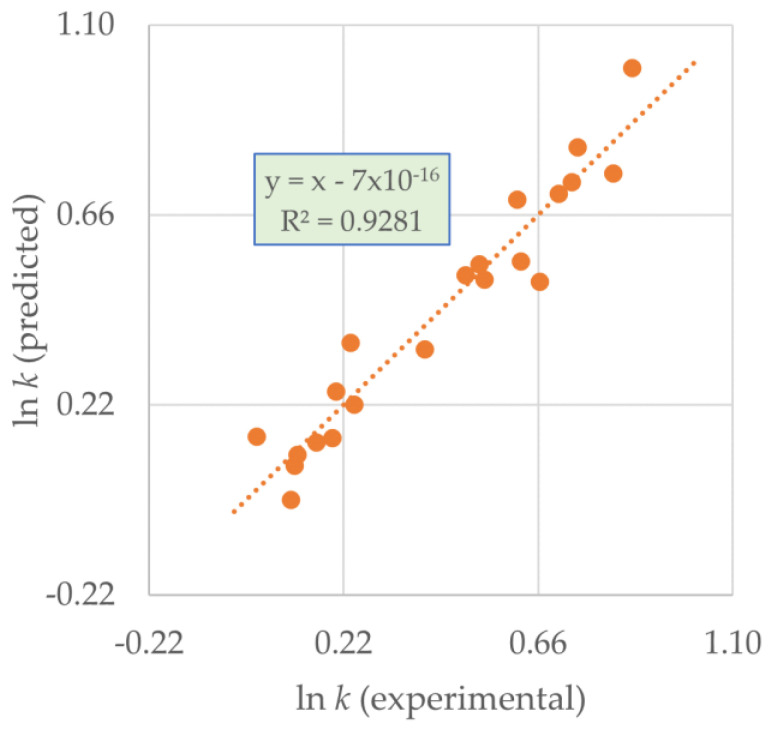
Predicted vs. experimental ln *k* for Equation (1).

Residuals are clearly randomly distributed around the trendline, as can be seen in Figure 4. The relative importance of the descriptors is:

*V*_vdW_ (−) > *ρ* (−) > [B]_pyr_ (+)

In line with our previous work, [B]_pyr_ is important and contributes here positively for ln *k*, although it is the least contributing descriptor, probably because a greater difference of Brønsted acidity among zeolites is needed in order to further enhance its importance. The major contributor, *V*_vdW_, which accounts for the volume occupied by the solute molecules, is detrimental toward *k*, showing that the size of the substrate dictates the ease of diffusion inside the zeolite pores. Additionally, the weight of this descriptor suggests that a large electron cloud establishes weaker bonding with the catalytic surface. Solute density, *ρ*, also has a negative role in the process, since a larger density causes steric hindrance and this, in turn, affects the ease of adsorption.

For *K*_r_, the relative equilibrium adsorption constant, the data point for the reaction of benzothiophene in the zeolite without treatment was rejected a priori because the obtained value is unrealistic. The best equation is:

(2) $$\begin{array}{ccccc} \ln K_{r}=\; & \left(0.31\;\pm \;0.02\right) & +\;\left(0.60\;\pm \;0.04\right)\sigma \; & +\;\left(0.45\;\pm \;0.04\right)\rho & \;-\;\left(0.06\;\pm \;0.02\right)V_{\mathrm{micro}}\\ & \left(100\%\right) & \left(100\%\right) & \left(100\%\right) & \left(99\%\right) \end{array}$$

*N* = 16; *R*^2^ = 0.963; *R*^2^_adj_ = 0.953; *R*^2^_pred_ = 0.935; *s_fit_* = 0.08; *F* = 103

In this case, there are four outliers due to a greater uncertainty in the calculation of *K*_r_. Again, only three descriptors are needed to conveniently model the adsorption/desorption behavior, and the best equation now includes *σ*, the solute’s surface area accessible to the zeolite, *ρ*, the solute’s density, and again, only one zeolite parameter, *V*_micro_. The relative importance of descriptors can be established as:

*σ* (+) > *ρ* (+) > *V*_micro_ (−)

and, therefore, we may say that the solute’s accessible surface area and the density increase *K*_r_, since desorption becomes more difficult due to the stronger adsorption. A larger microporous volume eases desorption relative to adsorption and thus, diminishes *K*_r_.

**Figure 5:**
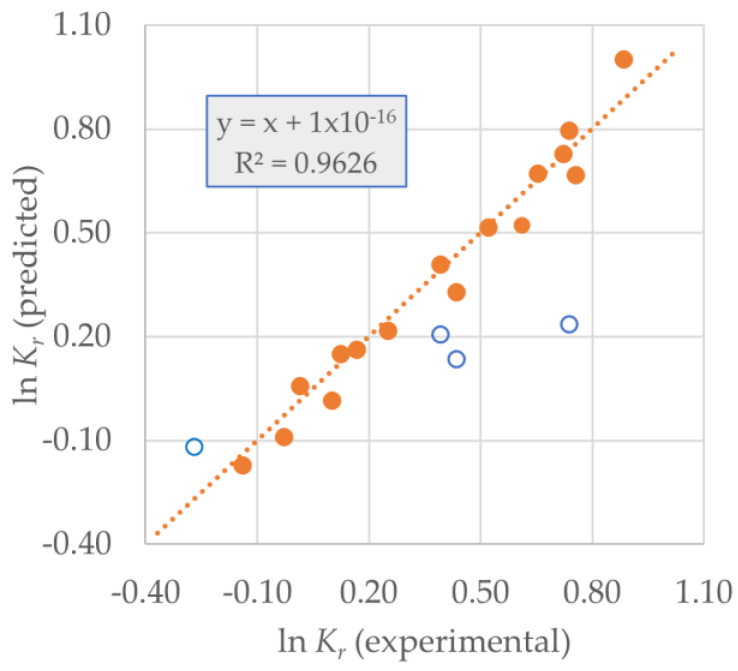
Predicted vs. experimental ln *K*_r_ for Equation (2) (◯ = outlier).

Figure 5 shows the fit for *K*_r_. A random distribution of data points along the fitted line can be observed.

Ideally, it would be very interesting to further assess the predictive ability of these model equations by estimating ln *k* and ln *K*_r_ for a different set of substrates not used in these correlations. This would imply the knowledge of values for these quantities, calculated in the same way for those “new” substrates, obtained in the same conditions, that is, for the same zeolite and the same acylating agent, at the same temperature. Unfortunately, to our knowledge, there are no such values available in the literature.

## 3. Materials and Methods

### 3.1. Materials and Chemicals

BEA zeolite with a Si/Al = 12.5 was supplied by Zeolyst (lot. CP 814E). The material was acquired as NH_4_BEA and converted into the protonic form by calcination under dry air (6 L h^−1^ g^−1^) at 500 °C for 3 h.

All chemicals used for zeolite modifications or reagents for Friedel-Crafts acylation reactions were purchased from Merck (Darmstadt, Germany) and were used as received, including the acylating agent, acetic anhydride, and all substrates, i.e., furan, anisole, pyrrole, benzofuran, thiophene, benzothiophene, and indole.

### 3.2. Post Synthesis Modifications of BEA Zeolite

The preparation of hierarchical BEA samples was performed using post synthesis procedures described in detail in our previous work [13]. In brief, the commercial BEA sample was treated with a 0.1 M NaOH (p.a.) solution (liquid-to-solid ratio = 30 mL g^−1^) at 60 °C for 30 min. Upon recovering by centrifugation, the solid was submitted to ion exchange with a 2 M NH_4_NO_3_ (p.a.) solution (liquid-to-solid ratio = 25 mL g^−1^) at 80 °C for 6 h, followed by calcination at 500 °C for 3 h under dry air. The desilicated samples were further submitted to an acid treatment using a 0.1 M HCl (p.a. fuming 37%) solution (liquid-to-solid ratio of 30 mL h^−1^) at 70 °C for 3 h. Upon recovering by centrifugation, the solid was calcined as described above. From now on, samples will be designated as BEA-D-AT, where “D” stands for the desilicated sample and “AT” is used in the case where acid treatment was applied.

### 3.3. Physicochemical Characterization

All zeolite samples were characterized through several techniques. The equipment and the procedures used are described in detail in a previous work [13]. Briefly, the structural characterization was obtained from X-ray powder diffraction (Philips, Almelo, The Netherlands), ^29^Si and ^27^Al MAS NMR spectroscopy (Bruker, Billerica, MA, USA), elemental analysis, and transmission electron microscopy (Hitachi, Chiyoda, Japan). The textural characterization was assessed through low temperature N_2_ adsorption assays and the acidity characterizations were studied by pyridine adsorption followed by FTIR spectroscopy (Nicolet, Thermo Fisher Scientific, Waltham, MA, USA).

### 3.4. Catalytic Experiments

For the catalytic assays, the amounts of each reactant were adjusted to keep the molar ratios between substrates and acylating agent equal to 5. As an example, a mixture of furan (0.71 g, i.e., 10.5 mmol) and acetic anhydride (5.4 g, i.e., 52.5 mmol) was added to a sample of parent or modified BEA zeolite (150 mg). The suspensions were placed in a three-necked round-bottom flask adapted with a reflux condenser, placed on a heating plate with temperature control (IKA C-MagHS7), under vigorous stirring. The suspensions were heated at 60 °C and periodically, small aliquots were withdrawn from the reaction mixture with a hypodermic syringe and filtered with a membrane filter (Millipore Swinnex support with a Millipore Durapore, 0.45 μm). The aliquots were quenched in an ice bath to “stop” the reaction, and then, injected in a gas chromatograph from a Perkin Elmer auto-system (Perkin Elmer, Norwalk, CT, USA) equipped with FID, using N_2_ as carrier gas and a 30 m DB-5MS capillary column (inner diameter of 0.32 mm and an I.D. of 0.25 μm) from Agilent to follow the reaction’s progress. All products were identified by comparison of retentions times with those of previously injected standard samples and the integration was made using DataApex CSW32 software 1.4 (DataApex, Prague, Czech Republic). In some cases, Capillary GC-MS analyses were performed on an Agilent 6890 Series gas chromatograph equipped with an Agilent 7683 automatic liquid sampler coupled to an Agilent 5973N mass selective detector (Agilent Technologies, Little Falls, DE, USA). A programmed temperature vaporization injector with a baffled liner and operating at 250 °C in the split mode (1:100) was used. The injection volume speed was set at 1 μL. GC-MS analysis was performed on a Zebron ZB-5 (30 m × 0.25 mm I.D., 0.25 μm df; Phenomenex, Torrance, CA, USA) capillary column (5% phenyl, 95% polydimethylsiloxane), using helium as carrier gas maintained in a constant inlet pressure mode of 7.81 psi. The oven temperature was programmed from 50 °C (5 min) at 20 °C/min to 160 °C (25 min) and finally, to 300 °C (1 min). The transfer line, ion source, and quadrupole analyzer temperatures were maintained at 280, 230, and 150 °C, respectively. In the full-scan mode, electron ionization mass spectra in the range 35–550 Da were recorded at 70 eV electron energy with an ionization current of 34.6 μA. Data recording and instrument control were performed by the MSD ChemStation software (G1701CA; version C.00.00; Agilent Technologies, Santa Clara, CA, USA).

### 3.5. Kinetic and QSPR Analysis

Kinetic modelling was based on the methodology already presented in detail in previous works [12,13]. Starting from the simple reaction scheme,

A_(ads)_ + S_(ads)_ → P_(ads)_

the deduction of an equation for the reaction rate, *r*, can be derived from the Langmuir-Hinshelwood model, leading to the following expression:

(3) $$r=\frac{k\;K_{A}K_{S}\left[A\right]\left[S\right]}{{\left(1+K_{A}\left[A\right]+K_{S}\left[S\right]+K_{P}\left[P\right]\right)}^{2}}\;$$

where A stands for the acylating agent, S for the substrate, and P for the acylated product; *k* represents the rate constant of the rate determining step, and *K*_A_, *K*_S_, and *K*_P_ are the adsorption equilibrium constants for each reagent and product, respectively.

It is possible to simplify Equation (3) through a sequence of approximations and logical steps, from which one obtains Equation (4) [13]:

(4) $$r≅\;\frac{k\;\left[A\right]\left[S\right]}{{\left(\left[A\right]+\left[S\right]+K_{r}\left[P\right]\right)}^{2}}$$

where *K*_r_ is the ratio between the adsorption equilibrium constant of the product and the normalized equilibrium constants of the reagents. The values of *k* and *K*_r_ as well as the corresponding statistical parameters are estimated through nonlinear regression using Table Curve2D^®^ software 5.0.1.

As described in previous articles [12,13], results can then be analyzed using a QSPR methodology. In QSPRs, a property, in this case ln *k* or ln *K*_r_, can be modelled as a linear combination of additive and (ideally) independent properties or descriptors, *D_i_*. Mathematically, we can express the models as multiple linear regression equations (MLR):

(5) $$\ln\;k\;(\mathrm{or}\;\ln\;K_{r})=a_{0}\;+\;\sum_{i=1}^{N}a_{i}D_{i}+\;\xi$$

where *ξ* is the associated model uncertainty, *a*_0_ is the regression-independent term, and *a_i_* are the regression coefficients associated with each descriptor.

The descriptors’ numerical values reflect the relative importance of the potentially relevant physicochemical properties which correlate with the observed property (ln *k* or ln *K*_r_) if one uses standardized independent variables. In the case of this work, since there is still scarce quantifiable knowledge about the relevance of specific descriptors, we gathered 14 descriptors either from the literature, measured in previous works, or calculated using MMPro Plus software (version 6.2.5), related to zeolite (7) and substrate (7) properties. These were then used to establish regression models with up to 5 independent variables as well as the correlation matrix of the parameters. All regressions with parameters showing a pairwise determination coefficient, *r*^2^, greater than 0.5 were discarded, since multicollinearity effects can distort MLR results. Additionally, a confidence interval of 95% was considered as the threshold to keep a given parameter in a regression.

Given the exceedingly high number of possible combinations of descriptors, an in-house conceived Visual Basic set of Macros for MS Excel^®^, which makes use of the Data Analysis ToolPack add-in, was employed to explore the whole independent variable space.

## 4. Conclusions

The inclusion of various substrates with different physicochemical characteristics, and the possibility to explore a larger number of properties/descriptors, led to more robust model equations that allow further insights into these Friedel-Crafts reactions.

Surprisingly, Lewis acidity, [L]_pyr_ has shown no significant role in the process, although it spanned for 31% against only 14% for Brønsted acidity, [B]_pyr_. Bearing in mind that [B]_pyr_ is strongly correlated with *V*_micro_, and *σ* is even more strongly correlated with *V*_vdW_, results indicate a somewhat inverse influence of similar parameters for (relative) equilibrium adsorption constants and rate constants: the same, or similar, parameters which decrease the rate constant also increase the difficulty in product desorption (larger *K*_r_). This can be rationalized in terms of stronger adsorption forces that, at the same time, increase catalytic efficiency but also make active sites less accessible to new molecules.

Results show that the most relevant parameter in both cases is related to substrate molecular size. However, these parameters may reflect different actions, namely accessibility to micropores, diffusion capacity of molecules through the zeolitic atomic network, and also molecular polarizability, factors that were already shown to be relevant in previous works using fewer and smaller model molecules but for which more support was now found due to the use of a larger set of increased size molecules.

Ultimately, we were able to quantitatively describe both rate and sorption equilibrium constants through two model equations, computed using Multiple Linear Regressions (MLR) and resorting to judicious statistical criteria.

## Figures and Tables

**Table 1:** Degree of crystallinity, *C*_XRD_, Si/Al ratio, textural parameters, and Brønsted, [B]_pyr_, and Lewis [L]_pyr_, acidic sites concentration [13].

Sample	*C*_XRD_^a^ (%)	Si/Al ^b^	*V*_micro_^c^ (cm^3^ g^−1^)	*V*_meso_^c^ (cm^3^ g^−1^)	*A*_ext_^c^ (m^2^ g^−1^)	[B]_pyr_^d^ (mmol g^−1^)	[L]_pyr_^d^ (mmol g^−1^)
BEA	100	12.5	0.17	0.40	246	0.225	0.273
BEA-D	75	8.3	0.11	0.44	247	0.203	0.254
BEA-D-AT	71	10.6	0.15	0.44	236	0.235	0.198

^a^ Calculated from powder X-ray diffraction patterns, using BEA12.5 as reference. ^b^ Total Si/Al ratio estimated from elemental analysis through ICP-OES spectrometry. ^c^ Textural parameters calculated from N_2_ adsorption isotherms: *V*_micro_ (micropore volume); *V*_meso_ (mesopore volume); and *A*_ext_ (external area). ^d^ Quantified from pyridine adsorption followed by FTIR, at 150 °C.

**Table 2:** Rate constants, *k*, and relative sorption equilibrium constants, *K*_r_, of the BEA catalytic reactions. Shown also are the fits’ statistical figures of merit ^a^.

	Furan *	Benzo- furan	Thiophene	Benzo- thiophene	Pyrrole **	Indole	Anisole **
*k* ^b^	65 ± 2	1.30 ± 0.03	97 ± 2	0.513 ± 0.002	17 ± 1	0.37 ± 0.04	13 ± 1
*K*_r_	8.0 ± 0.6	25 ± 2	7.1 ± 0.4	0.241 ± 0.006	20 ± 3	146 ± 50	54 ± 10
*R* ^2^	0.992	0.979	0.997	0.964	0.953	0.768	0.935
*s_fit_*	0.276	0.006	0.275	0.0001	0.162	0.007	0.152
*F*	1016	382	1866	190	144	23	86

^a^*R*^2^, determination coefficient; *s_fit_*, standard deviation of fit; *F*, Fisher-Snedecor statistics. ^b^ [*k*] ≡ mmol min^−1^ g^−1^. * Data from [13] updated with additional data points. ** Data from [13].

**Table 3:** Rate constants, *k*, and relative sorption equilibrium constants, *K*_r_, of the BEA-D catalytic reactions. Shown also are the fits’ statistical figures of merit ^a^.

	Furan *	Benzo- furan	Thiophene	Benzo- thiophene	Pyrrole **	Indole	Anisole **
*k* ^b^	58 ± 3	0.30 ± 0.01	82 ± 2	0.151 ± 0.007	16 ± 1	0.53 ± 0.05	3.0 ± 0.3
*K*_r_	12 ± 1	239 ± 29	5.8 ± 0.5	185 ± 31	26 ± 3	528 ± 195	84 ± 12
*R* ^2^	0.979	0.973	0.995	0.923	0.953	0.918	0.942
*s_fit_*	0.376	0.002	0.290	0.001	0.145	0.007	0.040
*F*	339	180	1345	60	144	57	98

^a^*R*^2^, determination coefficient; *s_fit_*, standard deviation of fit; *F*, Fisher-Snedecor statistics. ^b^ [*k*] ≡ mmol min^−1^ g^−1^. * Data from [13] updated with additional data point. ** Data from [13].

**Table 4:** Rate constants, *k*, and relative sorption equilibrium constants, *K*_r_, of the HBEA-D-AT catalytic reactions. Shown also are the fits’ statistical figures of merit ^a^.

	Furan *	Benzo- furan	Thiophene	Benzo- thiophene	Pyrrole **	Indole	Anisole **
*k* ^b^	164 ± 2	1.0 ± 0.1	783 ± 5	3.4 ± 0.5	11.4 ± 0.9	0.47 ± 0.03	11.9 ± 0.3
*K*_r_	14 ± 1	149 ± 44	28 ± 4	19 ± 8	21 ± 3	84 ± 19	40 ± 5
*R* ^2^	0.998	0.871	0.999	0.768	0.932	0.950	0.953
*s_fit_*	0.327	0.020	0.752	0.083	0.125	0.004	0.111
*F*	3870	27	18438	16	97	38	145

^a^*R*^2^, determination coefficient; *s_fit_*, standard deviation of fit; *F*, Fisher-Snedecor statistics. ^b^ [*k*] ≡ mmol min^−1^ g^−1^. * Data from [13] updated with additional data point. ** Data from [13].

**Table 5:** Substrate descriptors ^a^ used in this work.

		Substrate					
Descriptor	Thio-phene	Benzo- thiophene	Furan	Benzo-furan	Indole	Pyrrole	Anisole
*V*_vdW_ (cm^3^ mol^−1^)	42.775	68.943	61.712	64.18	63.009	37.658	64.37
*V*_m_ (cm^3^ mol^−1^)	78.064	124.23	57.523	105.12	100.11	55.294	107.96
*σ* × 10^9^ (cm^2^ mol^−1^)	5.3	7.806	4.688	7.311	7.157	4.762	8.189
*ρ* (g cm^−3^)	1.078	1.080	1.183	1.124	1.170	1.213	1.002
*μ* (D)	1.291	1.274	1.567	1.649	0.98	1.024	1.428
*η* (cP)	0.227	0.483	1.570	3.002	0.351	0.126	2.578
*γ* × 10^3^ (Nm^−1^)	34.65	32.141	58.970	43.205	52.762	76.263	36.528

^a^ Obtained from MMPro.

**Table 6:** Descriptors’ correlation matrix for the 14 descriptors used.

*r* ^2^	*V*_vdw_	*σ*	*ρ*	*V*_m_	*μ*	*η*	*γ*	*C*_XRD_	Si/Al	*V*_micro_	*V*_meso_	*A*_ext_	[B]_pyr_	[L]_pyr_
***V*_vdw_**	1	**0.96**	0.31	**0.95**	0.01	0.13	0.41	0.00	0.00	0.00	0.00	0.00	0.00	0.00
***σ***		1	0.45	**0.91**	0.01	0.19	0.43	0.00	0.00	0.00	0.00	0.00	0.00	0.00
***ρ***			1	0.43	0.11	0.15	**0.75**	0.00	0.00	0.00	0.00	0.00	0.00	0.00
***V*_m_**				1	0.01	0.09	**0.59**	0.00	0.00	0.00	0.00	0.00	0.00	0.00
***μ***					1	**0.67**	0.14	0.00	0.00	0.00	0.00	0.00	0.00	0.00
***η***						1	0.07	0.00	0.00	0.00	0.00	0.00	0.00	0.00
***γ***							1	0.00	0.00	0.00	0.00	0.00	0.00	0.00
***C*_XRD_**								1	**0.58**	0.44	**0.98**	0.29	0.01	**0.61**
**Si/Al**									1	**0.98**	**0.70**	0.02	**0.51**	0.04
***V*_micro_**										1	**0.57**	0.07	**0.64**	0.00
***V*_meso_**											1	0.18	0.04	0.48
***A*_ext_**												1	**0.63**	**0.90**
**[B]_pyr_**													1	0.31
**[L]_pyr_**														1

Values of the determination coefficients, *r*^2^, higher than 0.5 are presented in bold.
